# Bone marrow mesenchymal stem cells attenuate LPS-induced acute lung injury in mice by promoting RvE1/ProD1 and modulating Treg/Th17 balance

**DOI:** 10.3906/biy-2107-83

**Published:** 2021-12-20

**Authors:** He-bu QIAN, Guijuan ZOU, Chao LI, Qi-fang HE, Jun LIU

**Affiliations:** 1 Department of Critical Care Medicine, Suzhou Ninth People’s Hospital, The Affiliated Wujiang Hospital of Nantong University, Suzhou, China; 2 Gusu School of Nanjing Medical University, Department of Critical Care Medicine, The Affiliated Suzhou Hospital of Nanjing Medical University, Suzhou Municipal Hospital, Suzhou Clinical Medical Center of Critical Care Medicine, Suzhou, China

**Keywords:** Acute lung injury, acute respiratory distress syndrome, mesenchymal stem cells, proresolving mediators, Treg, Th17 cells

## Abstract

Acute lung injury (ALI) and its severe form acute respiratory distress syndrome (ARDS) are respiratory failures caused by excessive alveolar inflammation with high mortality. In this study, we investigated the effects of bone marrow mesenchymal stem cells (BMSCs) on lung injury of lipopolysaccharide (LPS)-induced ALI and explored the associated mechanisms. BMSCs were isolated, cultured, identified by staining with CD34 and CD44 surface markers. LPS-induced ALI mouse model was generated by injecting with LPS and divided into ALI group and ALI+BMSCs group. Mice treated without any reagents were assigned as Control, mice transplanted with BMSCs were assigned as BMSCs group. Regulatory T (Treg) and Th17 percentages were evaluated using flow cytometry. Proresolving mediators (resolvin E1 (RvE1), protectin D1 (ProD1)) in lung tissue and cytokines (interleukin-6 (IL-6) and IL-17) in serum were analyzed by ELISA. Myeloperoxidase (MPO) activity was determined. Cultured cells demonstrated typical characteristics of BMSCs. BMSCs transplantation (ALI+BMSCs) obviously alleviated LPS-induced ALI in mice. BMSCs transplantation significantly decreased MPO activity in LPS-induced ALI in mice compared to the Control group (p < 0.05). BMSCs transplantation markedly increased Treg percentages and decreased dendritic cells (DCs) and Th17 cells percentages compared to those of the Control group (p < 0.05). BMSCs transplantation remarkably enhanced RvE1 and ProD1 levels in LPS-induced ALI (ALI+BMSCs) compared to the ALI group (p < 0.05). BMSCs transplantation significantly attenuated IL-6 and IL-17 levels in serum of mice treated with LPS (ALI+BMSCs) compared to those of the ALI group (p < 0.05). In conclusion, BMSCs transplantation effectively attenuated LPS-induced pathological injury of ALI in mice, at least partly through promoting proresolving mediators RvE1 and ProD1 and modulating the balance of Treg/Th17.

## 1. Introduction

Acute lung injury (ALI) and its severe form- acute respiratory distress syndrome (ARDS) is the respiratory failure in critically ill patients resulted from excessive alveolar inflammation (Matthay et al., 2019). Despite the recent advances in lung protective ventilation strategies, overall hospital mortality for ARDS patients was 34.0%, with 60.0% patients diagnosed as severe ARDS (Liu et al., 2018). Lack of effective pharmacological treatment remains the main reason for high mortality of ARDS.

Imbalance of inflammatory response plays a key role in occurrence and development of ALI, which makes the pathogenesis extremely complex (Englert et al., 2019). Among various immune cell types, CD4^+^ T cells play an important part in the pathogenesis of ALI (Adamzik et al., 2013; Weiskopf et al., 2020). Regulatory T (Treg) cells have antiinflammatory roles mainly caused by contact-dependent suppression and release of cytokines such as transforming growth factor β (TGF-β) and interleukin-10 (IL)-10 that negatively impact other effector immune cells (Weaver and Hatton RD, 2009). In contrast, Th17 cells play a strong pro-inflammatory role through production and release of cytokines, including IL-17, IL-6, tumor necrosis factor α (TNF-α). Imbalance of Th17/Treg has been found in a number of different autoimmune and inflammatory diseases (Weaver and Hatton RD, 2009). A recent prospective and observational study has demonstrated that the ratio of Th17/Treg cells was positively related to disease severity and 28-day mortality in patients with ARDS (Yu et al., 2015). Furthermore, our previous data with lipopolysaccharide (LPS)-induced ALI in mice also demonstrated that lung dendritic cells (DCs) activation and Th1/Th17 polarization may play an important role in the pathogenesis of ALI (Liu et al., 2012a; Liu et al., 2012b). Therefore, strategies designed to restore Th17/Treg balance may be a novel and effective therapeutic strategy in ARDS.

Mesenchymal stem cells (MSCs) have a wide range of tissue sources and demonstrate a strong ability of self-renewal and multi-directional differentiation (Islam et al., 2019). A recent study (Qin and Zhao, 2020) has demonstrated that MSCs can ameliorate lung injury in preclinical ARDS models, but the associated mechanisms remain not fully understood. Data of MSCs on differentiation of naive T cells into T cells subtypes in ALI remain scarce. A recent study (Chen et al., 2020) observed that MSCs could induce the development and differentiation of Treg cells and modulate Th17/Treg balance. However, modulation of MSCs on Th17/Treg balance in ALI remains largely elusive.

The resolution of inflammation is an active course that limits inflammation and prevents collateral injury. The resolution of inflammation is mediated by the specialized proresolving lipid mediators (SPMs), including lipoxins, resolvins, and protectins (Fredman, 2019). This process seems to be defective in several common inflammatory lung diseases that are characterized by excessive inflammation, such as ARDS (Levy and Serhan, 2014). Recently, Gagliani et al. (2015) demonstrated that Th17 cells trans-differentiation into Treg cells may contribute to the resolution of inflammation. Using single-cell RNA-sequencing, Sharma, et al. (Sharma et al., 2020) reported that Treg cells can produce specialized proresolving lipid mediators and enhance inflammation resolution in mouse models of atherosclerosis. Treg cells may tune the regulation of tumor inflammation through lipoxin A4 (Zhang et al., 2010). However, the regulatory effect of SPMs on the balance of Th17/Treg remains unknown in ALI. Therefore, we hypothesized that bone marrow-derived mesenchymal stem cells (BMSCs) might affect lung injury through promoting SPMs and modulating Th17/Treg balance in ALI. In this study, we aimed to construct an LPS-induced ALI mouse model by intratracheally administrating mice with LPS and to investigate effects of BMSCs on expressions of SPMs and immune cells in lung tissues of mice treated with LPS. The present study would provide a theoretical basis for the application of BMSCs in treating ALI clinically.

## 2. Materials and methods

### 2.1. Study design

After a week of adaptive feeding, LPS-induced ALI mouse model was established. One week after modeling, BMSCs were injected into tail vein. Twenty-four hour and 72h after BMSCs injection, lung tissues of mice in each group were collected (and homogenized) for flow cytometry assay to detect the proportion of DCs, Th17, and Treg cells. ELISA was used to detect contents of lipoxin A4, regressin E1 and protectin D1 in lung tissues, and hematoxylin and eosin (H&E) staining was used to detect pathology of lung tissues. At the same time, blood samples were also collected for ELISA to detect serum levels of IL-17 and IL-6.

### 2.2. Isolation and culture of BMSCs

In the present research, BMSCs were isolated as described by a former study (Chen et al., 2018), with a few modifications. Briefly, BMSCs were isolated from the bone marrow of femur and tibia deriving from C57BL/6 mouse (Experimental Animal Center of Nanjing Medical University, Nanjing, China), using density-gradient centrifugation separating method with Ficoll-Hypaque (GE Bioscience, NJ, USA). The obtained cells were washed, suspended using Dulbecco’s modified eagle medium (DMEM, Gibco BRL. Co. Ltd., Grand Island, New York, USA), and cultured with DMEM containing 15% fetal bovine serum (FBS, Gibco BRL. Co. Ltd.) and 1% streptomycin-penicillin (Beyotime Biotech., Shanghai, China) at 37 °C and 5% CO_2_ for 24h. When the cultured cells achieved a confluence of 80%, they were digested using 0.25% trypsin (Beyotime Biotech.) and passaged for 3 generations. The morphology for isolated BMSCs was observed with an inverted microscope (Model: IX73, Olympus, Tokyo, Japan). All processes for animal experiments or tests were in accordance with NIH guidelines and have been approved by the IACUC of Nanjing Medical University.

### 2.3. Identification for BMSCs

The thirdly passaged cells were adjusted to density of 2×10^6^ cells/mL medium, washed using phosphate buffer solution (PBS), and stained with fluorescence-conjugated antibodies specific for phycoerythrin (PE)-labeled CD44 and FITC-labeled CD34 (eBioscience, Santiago, CA, USA) in dark and on ice for 20 min. Then, cells were washed using PBS (supplementing with 1% FBS) for 2 times and re-suspended with FACS Canto II flow cytometer (BD Biosciences, Franklin Lakes, NJ, USA). The BMSCs were negative for CD34 and positive for CD44.

### 2.4. Generation for LPS-induced ALI mouse model and grouping

A total of 24 C57BL/6 mice, aging 6–8 weeks and weighting 20–25 g, were randomly divided into 4 groups (6 mice each group), including the Control group, BMSC group, ALI model group, and ALI+BMSCs group. Mice in control were only injected with normal saline in airway. Mice in BMSC group were injected with normal saline in airway and 30 min later injected with BMSCs (10^6^ cells per mouse) into tail veins. Mice in ALI group were injected with LPS (5 mg/ml storage solution) at final dosage of 1 μL/g body weight into airway and without BMSCs injection. Mice in ALI+BMSC group were injected with LPS (5 mg/ml storage solution) at final dosage of 1 μL/g body weight into airway and 30 min later injected with BMSCs (10^6^ cells per mouse) into tail veins.

### 2.5. Lung injury assessment

Severity of lung injury was measured using lung tissue histopathology and lung injury scores as previously described (Liu et al., 2012a; Liu et al., 2012b). Mice were anesthetized by intraperitoneally injecting with chloral hydrate (0.5 mL**/**100 g body weight) and sacrificed. Lung tissues of mice were stained using H&E method for carrying out histopathological analysis. Briefly, the newly separated lung tissues were fixed using 4% paraformaldehyde (Beyotime Biotech.) at room temperature for 30min. Then, lung tissues were dehydrated and embedded in paraffin prior following cutting into section of 5 μm thickness. Sections were stained using hematoxylin (10min, Jiancheng Bioengineering Institute, Nanjing, China) and eosin (30s, Beyotime Biotech.), as reported routine staining protocol everywhere, and analyzed with a microscope (Model: XC31, Olympus, Tokyo, Japan). A pathologist blind to the experimental groups semiquantitatively assessed the severity of lung injury (lung injury scores) according to our previously published studies (Liu et al., 2012a; Liu et al., 2012b).

### 2.6. Measurement for myeloperoxidase (MPO) activity in lung tissues

MPO activity assay, an index of neutrophil infiltration in pulmonary tissue, was carried out as descriptions of a former study (Dong et al., 2012). In brief, MPO activity was measured with myeloperoxidase assay kit (Model: A044-1-1, Jiancheng Bioengineering Institute, Nanjing, China) as instructed by manufacturer. MPO activity was calculated using following formula: MPO activity (U/g tissue wet weight) = (OD_measurement_-OD_control_)/(11.3^a^× sampling amounts (g)^b^), among which “a” represented reciprocal of slope, “b” represented amounts of wet tissues in samples.

### 2.7. Bronchoalveolar lavage fluid (BALF) collection and methylene blue-eosin staining

BALF of mice was collected as described by Wei-xu et al. (Wei-xu et al., 2014; Wei-xu et al., 2016). Briefly, BALF was collected at 24h and 72h post BMSCs injection. The left trachea was exposed firstly then was cannulated using silicone tubing attached to a needle (about 23 gauge) on a syringe with a volume of 5 mL. Postinstillation of sterile PBS (2 mL) through the trachea into lung tissues, BALF was withdrawn and centrifuged. Finally, supernatants were collected for following methylene blue-eosin staining.

Methylene blue-eosin staining was carried out as reported by Wei-xu et al. (Wei-xu et al., 2014). Briefly, smears of BALF were placed on the staining rack, 3–5 drops of methylene blue (Nanjing Jiancheng Biotech., Nanjing, China) were added, and stained about 1min to fix the smears. Then, smears were stained by directly adding 6–10 drops of eosin (Nanjing Jiancheng Biotech.), gently shaking glass slide to mix dye solution, and dying for 5 min. Finally, smears were washed with water for 60 s. After drying, cells on smears could be examined microscopically. The cytoplasm was red, and the nucleus was blue or purple.

### 2.8. Flow cytometry evaluation for dendric cells (DCs), Treg and Th17 cells

Isolation of lung single cells was conducted as reported previously (Liu et al., 2012a; Liu et al., 2012b). The DCs, Treg, and Th17 cells were analyzed with flow cytometry as reported previously (Liu et al., 2012a; Liu et al., 2012b). In brief, the single lung cells were fixed with 4% paraformaldehyde (Beyotime Biotech.) at room temperature for 30 min and permeabilized for 10 min. The cells were washed using PBS and stained with FITC-labeled anti-CD11c antibody (N418, Cat. No. 42-0114-81) and PE-labeled MHC-II antibody (AF6-120.1, Cat. No. 12-5320-80) for analyzing DCs, using FITC-labeled anti-CD3 antibody (UCHT1, Cat. No. 22-0304-72), APC-Cy7-labeled anti-CD4 antibody (GK1.5, Cat. No. A15384) and PE-labeled IL-17A antibody (eBio64DEC17, Cat. No. 12-7179-41) for analyzing Th17 cells and using FITC-labeled anti-CD4 antibody (GK1.5, Cat. No. 11-0041-82), PE-labeled anti-Foxp3 antibody (FJK-16s, Cat. No. 12-5773-82) and APC-labeled CD25 antibody (CD25-3G10, Cat. No. MHCD2505) for analyzing Treg cells overnight in dark at 4 °C. All these antibodies were purchased from eBioscience (Santiago, CA, USA). Eventually, flow cytometry was conducted on FACS Canto II flow cytometer (BD Biosciences) and the percentages of DCs, Th17, and Treg cells were evaluated with CELIQUEST software 5.1 (Biosciences, Franklin Lakes, NJ, USA).

### 2.9. ELISA for determining SPM and cytokines

In this study, the inflammation-associated factors in lung tissues, including lipoxin A4 (LXA4), resolvin E1 (RvE1), protectin D1 (ProD1), were measured. Meanwhile, the cytokines in serum, including IL6 and IL-17, were also measured using ELISA Kits (LkcxTech., Beijing, China) as instructed by the manufacturers.

### 2.10. Statistical analysis

Data were assigned as mean ± standard deviation (SD) and analyzed with SPSS software (version: 20.0, IBM Corp., Armonk, NY, USA). Differences among data of different groups were analyzed using post hoc Turkey validated ANOVA analysis. The p < 0.05 was assigned as significant difference.

## 3. Results

### 3.1. Culture and identification of BMSCs

**Figure 1:**
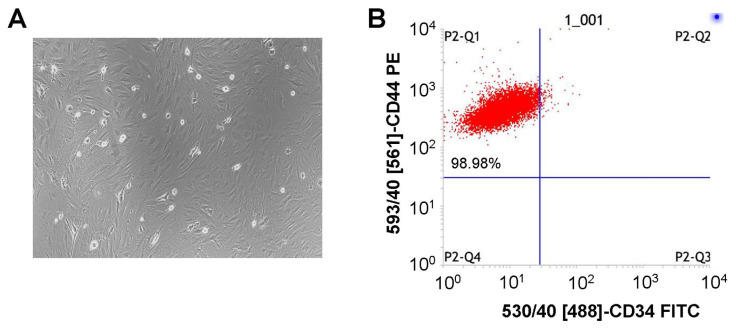
Culture and identification of BMSCs. A. Morphological characteristics for isolated and cultured cells in the DMEM medium. B. Identification and phenotypic analysis for BMSCs by staining with the surface biomarkers of CD44 and CD34.

After primary culture for 24h, the number of cells was small and fusiform. When the third generation was passaged, the number of cells increased gradually, and cells grew in a colony like vortex (Figure 1A). The flow cytometry results showed that a surface biomarker of BMSCs, CD44, was positively expressed with a rate of 98.98% (Figure 1B). Similarly, we found that BMSCs do not express the surface biomarker CD34 (Figure 1B). The cultured cells demonstrated typical characteristics of BMSCs, therefore, the isolated cells were identified as BMSCs.

### 3.2. BMSCs transplantation alleviated LPS-induced ALI in mice

**Figure 2:**
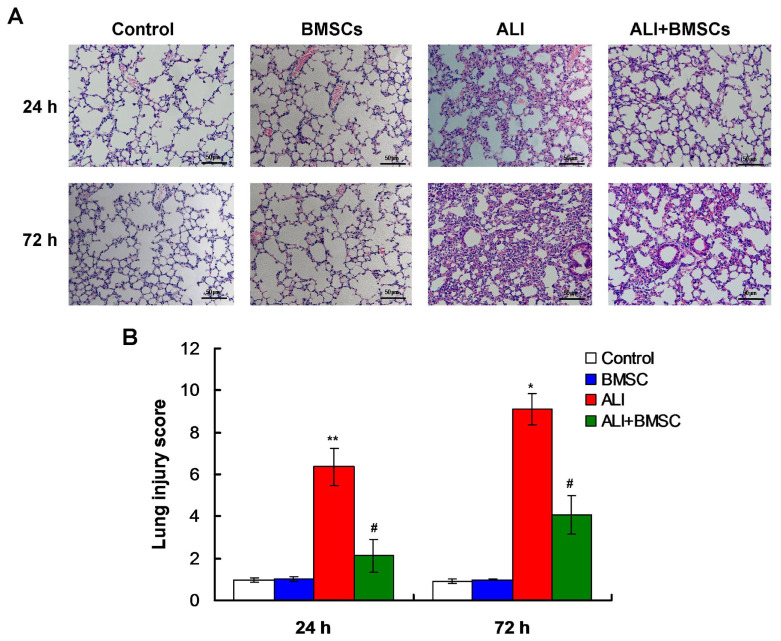
Lung tissue morphology of mice determined by the H&E staining. A. The H&E staining images for the alveolar structure of lung tissue in mice of different groups. B. Statistical analysis for the H&E staining. Magnification, 400×.

Under a light microscope, the alveolar structure of Control group and BMSC group was clear, and the alveolar septum was complete (Figure 2A) at both 24h and 72h of culture. In ALI group, the normal structure of the alveolar disappeared, the alveolar septum significantly thickened with extensive formed hyaline membrane and some inflammatory cells infiltrated at both 24h and 72h (Figure 2A). In ALI+BMSC group, alveolar structure was basically clear, alveolar septum was slightly thickened, and only a few inflammatory cells infiltrated into the pulmonary interstitium at both 24h and 72h (Figure 2A). Similarly, as shown in Figure 2B, lung injury scoring in ALI group was significantly higher than that in control mice at 24h and 72h (p *<* 0.01). Treatment of ALI group with BMSCs significantly decreased lung injury score, compared with that of ALI group (Figure 2B, p *<* 0.05). Totally, the transplantation of BMSCs into airway could alleviate LPS-induced ALI in mice

### 3.3. BMSCs transplantation decreased MPO activity in LPS-induced ALI in mice

**Figure 3:**
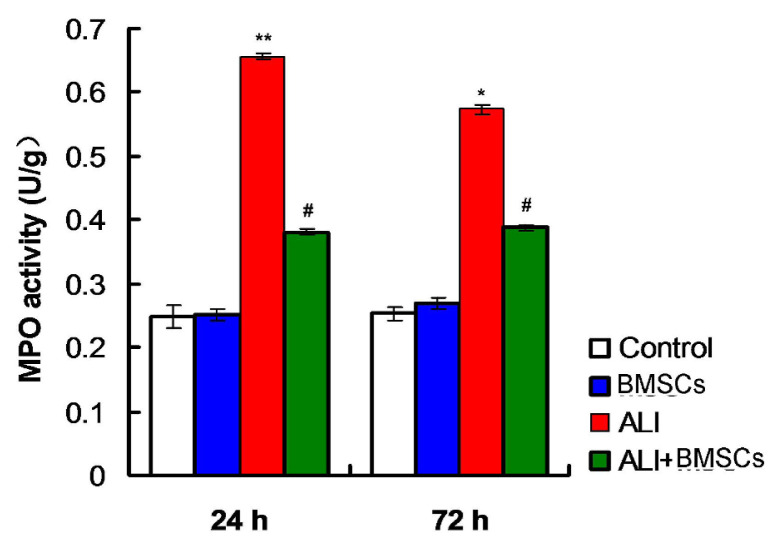
The statistical analysis for the MPO activity of lung tissues of mice. The MPO activity was measured using the Myeloperoxidase Assay Kit. ^*^p < 0.05 vs. ALI or ALI+BMSCs group. ^#^p < 0.05 vs. ALI group.

The results indicated that MPO activity in lung tissues of mice treated with LPS (ALI group) was significantly higher compared to those of the Control group mice and mice in BMSC group at 24h posttreatment (Figure 3, p *<* 0.01). However, MPO activity was significantly decreased in lung tissues of mice in ALI+BMSC group compared to those in ALI group at 24h posttreatment (Figure 3, p *<* 0.05). Moreover, at 72h post LPS or normal saline treatment, BMSCs transplantation (ALI+BMSC group) could also remarkably reduce MPO activity compared to that in ALI group (Figure 3, p *<* 0.05). Therefore, BMSCs transplantation could decrease MPO activity in LPS-induced ALI in mice

### 3.4. BMSCs transplantation reduced neutrophils in BALF of mice with LPS-induced ALI

**Figure 4:**
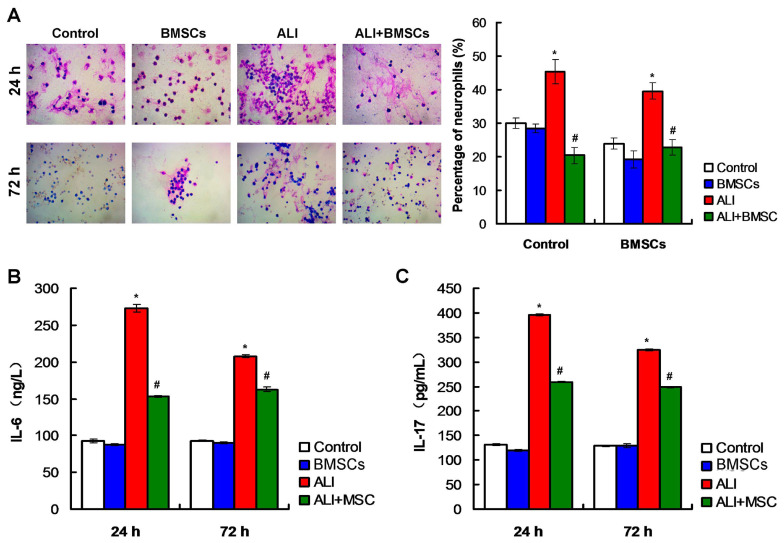
Effects of BMSCs transplantation on neutrophils in bronchoalveolar lavage fluid (BALF) and on serum levels of IL-6 and IL-17. A. Detection and statistical analysis for neutrophils in BALF using methylene blue-eosin staining. B. The levels of levels of IL-6 in the serum of mice were decreased by ELISA. C. The levels of IL-17 in the serum of mice were detected by ELISA. ^*^p < 0.05 vs. ALI or ALI+BMSCs group. ^#^p < 0.05 vs. ALI group.

The methylene blue-eosin staining findings showed that there were fewer neutrophils in BALF of mice in Control group and BMSCs group, at 24h and 72h post LPS treatments (Figure 4A). While there were more neutrophils in BALF of mice with LPS-induced ALI, BMSCs transplantation obviously reduced the percentage of neutrophils in BALF of mice (Figure 4A, p *<* 0.05). Thus, transplantation of BMSCs could reduce the number of neutrophils in BALF of mice with LPS-induced ALI.

### 3.5. BMSCs transplantation attenuated serum IL-6 and IL-17 levels in mice with LPS-induced ALI

Compared to Control and BMSC group, IL-6 level (Figure 4B) and IL-17 level (Figure 4C) were significantly increased in serum of mice in ALI group (p *<* 0.05), at 24h and 72h post LPS treatments. However, the treatment of BMSCs (ALI+BMSC group) could markedly attenuate IL-6 level (Figure 4B) and IL-17 level (Figure 4C) compared with those in serum of mice in ALI group (p *<* 0.05). These results suggest that BMSCs transplantation could inhibit the release of inflammatory cytokines such as IL-6 and IL-17 in mice with LPS-induced ALI.

### 3.6. BMSCs increased the percentage of Treg cells and decreased the percentage of DCs and Th17 cells in mice with LPS-induced ALI

**Figure 5:**
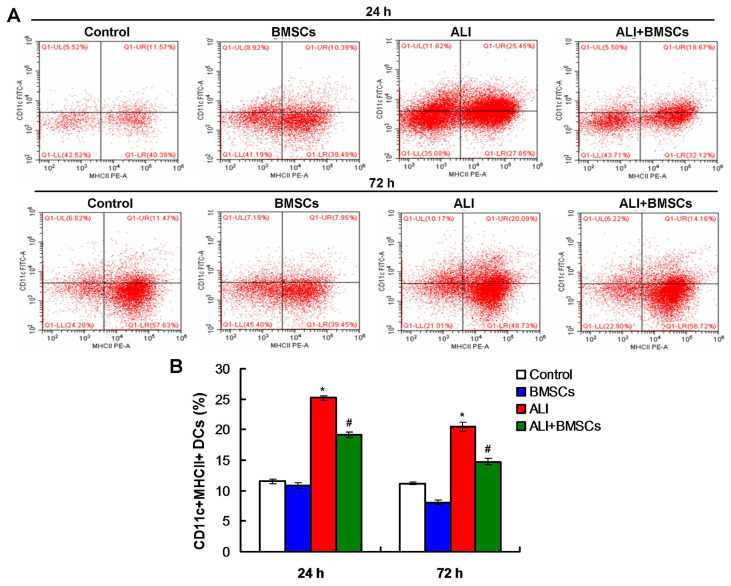
Effects of BMSCs transplantation on the percentage of DCs in mice with LPS-induced ALI. A. Flow cytometry plots for DCs at 24h and 48h posttreatments. B. Statistical analysis of DCs cells. ^*^p < 0.05 vs. ALI or ALI+BMSCs group. ^#^p < 0.05 vs. ALI group.

**Figure 6:**
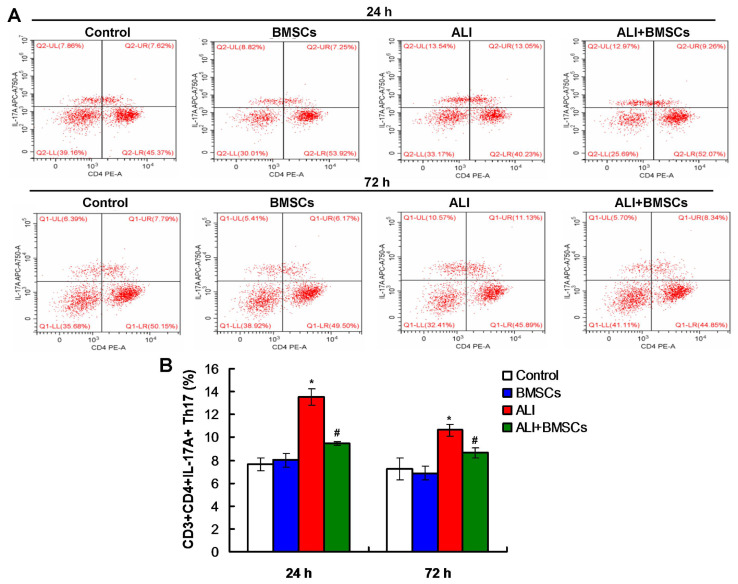
Effects of BMSCs transplantation on the percentage of Th17 in mice with LPS-induced ALI. A. Flow cytometry plots for Th17 cells at 24h and 48h posttreatments. B. Statistical analysis of Treg cells. ^*^p < 0.05 vs. ALI or ALI+BMSCs group. ^#^p < 0.05 vs. ALI group.

**Figure 7:**
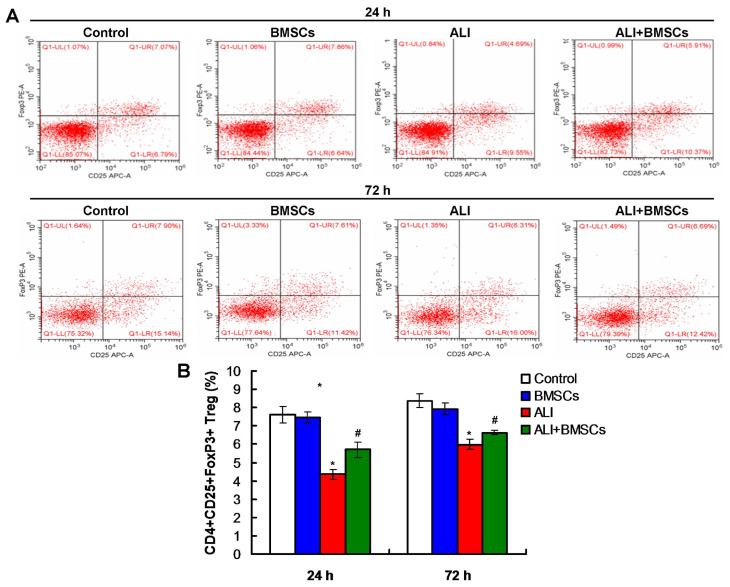
Effects of BMSCs transplantation on percentage of Treg in mice with LPS-induced ALI. A. Flow cytometry plots for Treg cells. B. Statistical analysis of the percentages of the Treg cells at 24h and 72h posttreatments. ^*^p < 0.05 vs. ALI or ALI+BMSCs group. ^#^p < 0.05 vs. ALI group.

Flow cytometry assay was used to detect the percentage of DCs, Treg, and Th17 cells. According to flow cytometry plots for DCs (Figure 5A), the percentage of DCs in mice of ALI group was remarkably increased compared to those of the Control group and BMSCs group (Figure 5B, p *<* 0.05) at 24h post LPS treatment. BMSCs (BMSC+ALI) markedly decreased the percentage of DCs than in mice of the ALI group (Figure 5B, p *<* 0.05), at 24h post LPS and/or BMSCs treatment. Also, according to flow cytometry plots (Figure 6A), BMSCs (BMSC+ALI) significantly reduced the percentage of Th17 cells in mice compared to those in ALI group, at 72h post LPS and/or BMSCs treatment (Figure 6B, p *<* 0.05). Moreover, flow cytometry plots (Figure 7A) also demonstrated that BMSCs (BMSC+ALI) significantly increased the percentage of Treg cells in mice compared to those in ALI group, at 72h post LPS and/or BMSCs treatment (Figure 7B, p *<* 0.05). Therefore, BMSCs could increase the percentage of Treg cells and decrease the percentage of DCs and Th17 cells.

### 3.7. BMSCs transplantation modulated RvE1 and ProD1 in lung tissues of LPS-induced ALI in mice

**Figure 8:**
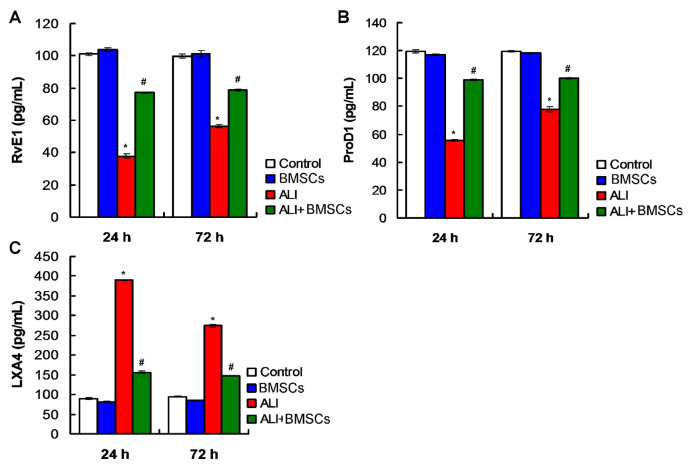
Effects for BMSCs transplantation on the serum levels of RvE1 (A) and ProD1 (B) of mice in different groups at 24h and 48h posttreatments. ^*^p < 0.05 vs. ALI or ALI+BMSCs group. ^#^p < 0.05 vs. ALI group.

In this study, proresolving lipid mediators, including RvE1, ProD1, and LXA4, have been determined using ELISA method. The results exhibited that RvE1 (Figure 8A) and ProD1 levels (Figure 8B) were significantly lower, and LXA4 level (Figure 8C) was significantly higher in lung tissues of mice with LPS-induced ALI compared to those in Control and BMSCs group (p *<* 0.05), at both 24h and 72h post LPS treatments. Interestingly, BMSCs (ALI+BMSC group) remarkably enhanced RvE1 (Figure 8A) and ProD1 levels (Figure 8B) compared to mice in ALI group (p *<* 0.05). BMSCs transplantation (ALI+BMSC group) significantly decreased LXA4 level (Figure 8C) in mice compared to those in ALI group (p *<* 0.05). These results suggest that BMSCs transplantation could modulate the production of RvE1 and ProD1 in lung tissues of mice with LPS-induced ALI.

## 4. Discussion

BMSCs show multi-directional differentiation potential and immuno-regulation function, therefore, BMSCs are widely applied in basic research for various inflammatory diseases treatment, such as ALI (Chen et al., 2020). Previously, we and other investigators have found that activation of conventional DCs and Th17 polarization may play an important role in the pathogenesis of ALI (Liu et al., 2012a; Liu et al., 2012b; Verjans et al., 2013; Yu et al., 2015), while Treg may play a protective role in ALI (D’Alessio et al., 2009; Adamzik et al., 2013). In the current study, we found that the treatment of BMSCs obviously alleviated lung injury in mice with LPS-induced ALI. Moreover, BMSCs treatment caused a significant increase in the frequency of Treg cells and a decrease in the percentage of Th17 cells and relevant proinflammatory cytokines. We further provided evidence that BMSCs transplantation markedly increased RvE1 and ProD1 levels in lung tissues of mice with LPS-induced ALI. These results suggest that the protective effects of BMSCs on ALI may be dependent, at least in part, on the promotion of proresolving mediators RvE1 and ProD1 and on the modulation of Th17/Treg balance.

In this study, BMSCs were obtained from bone marrow and identified by microscopic examination and flow cytometry. The results showed that the isolated cells have typical BMSCs morphology and molecular phenotype. Among all MSCs, BMSCs have been proven to demonstrate stronger anti-inflammatory ability compared to the other MSCs (Yang et al., 2018). Consistent with many previous reports (Chen et al., 2018; Yang et al., 2018; Islam et al., 2019; Chen et al., 2020; Qin and Zhao, 2020), we observed that the integrity of alveolar in mice with LPS-induced ALI was significantly improved, and inflammatory cells were significantly reduced after intervention of BMSCs. These results confirmed that the treatment of BMSCs partially prevented lung inflammatory process and lung injury in mice with LPS-induced ALI. Further investigations are needed to elucidate potentially protective mechanisms of BMSCs in ALI.

CD4^+^ T cells play an important part in the pathogenesis of ALI (Adamzik et al., 2013; Weiskopf et al., 2020). Some previous studies (Weaver and Hatton RD, 2009; Adamzik et al., 2013; Yu et al., 2015; Weiskopf et al., 2020) have reported that imbalance of Th17/Treg was correlated to inflammatory response and the severity of ALI. BMSCs might display strong immuno-regulation ability. In the present study, our results indicated that BMSCs transplantation remarkably enhanced the percentage of Treg cells and decreased the percentage of Th17 cells, both of which might attenuate inflammatory responses of mice with LPS-induced ALI. Therefore, BMSCs transplantation demonstrated remarkable antiinflammatory effects on LPS-induced ALI in mice through modulating the balance of Treg and Th17 cells. Eventually, Th17/Treg balance was re-generated in the lung tissues. A more recent study (Chen et al., 2020) also reported that BMSCs administration could reduce levels of proinflammatory cytokines and influence the balance of Th17/Treg, which are consistent with the present findings.

The acute inflammatory response is a complex process including three phases: inflammation, normal resolution, and postresolution phase (Fullerton and Gilroy DW, 2016). It has been recognized that resolution of inflammation is an active course that is initiated in the first few hours after an inflammatory process begins (Fullerton and Gilroy DW, 2016). Meanwhile, the resolution of inflammation is also mediated by SPMs, including lipoxins, resolvins, and protectins (Fullerton and Gilroy DW, 2016). These proresolving molecules promote the clearance of inflammatory cells from tissue site and promote the restoration of functional homeostasis (Leslie, 2015; Fullerton and Gilroy DW, 2016). The key histological character in the resolution of inflammation is loss of polymorphonuclear neutrophils from the local inflammatory sites (Serhan et al., 2008). Our study showed that BMSCs transplantation can significantly reduce MPO activity in lung tissue of mice with LPS-induced ALI. These findings suggest that BMSCs might have an important role in proresolution of lung inflammation of ALI.

The proresolving mediators, RvE1, ProD1, could inhibit inflammatory cell infiltration and suppress injury of tissues (Serhan et al., 2008; Leslie, 2015; Fullerton and Gilro, 2016). Therefore, the increased production of RvE1 and ProD1 would be beneficial to the reduction of inflammation. Zhang et al. (Zhang et al., 2020) proved that RvE1 played anti-inflammatory functions in LPS-induced heart injury. Duffield et al. (Duffield et al., 2006) reported that ProD1 protected against inflammatory responses of acute kidney injury in an animal model. However, the potential roles for RvE1 and ProD1 have not been clarified in LPS-induced ALI animal model to our knowledge. We further analyze several main SPMs to clarify potential proresolution effect of BMSCs in LPS-induced ALI in mice. In our study, we found that BMSCs transplantation remarkably enhanced expressions of antiinflammatory molecules, RvE1, and ProD1, in lung tissues of mice with LPS-induced ALI. Actually, proresolving mediators are usually produced in process of apoptotic cells clearance by the immune system of the body (Schwab et al., 2007). Therefore, we speculated that the enhancement of proresolving mediators, RvE1, ProD1 was also triggered by BMSCs through modulating immune responses. Moreover, to our knowledge, this is the first study that verifies proresolving mediators in the pathogenic process of ALI in an animal model. However, the cause-and-effect relationship between SPMs and Th17/Treg balance in ALI has not been firmly established. Tregs may produce SPMs and enhance inflammation resolution in mouse models of atherosclerosis and tumor (Zhang et al., 2010; Sharma et al., 2020). On the other hand, a more recent study (Chiurchiù et al., 2016) observed that SPMs, including resolvin D 1 (RvD1), resolvin D2 (RvD2), and maresin 1 (MaR1), can exert an important regulatory role on Th17 and Treg cells. Thus, we can not exclude the possibility that SPMs and Treg cells may generate a positive feedback loop, resulting in promotion of inflammation resolution in ALI treated with BMSCs. Further research is needed to clarify this relationship between SPMs and Th17/Treg cells.

This study also demonstrated a few limitations. First, this study only identified the Treg and Th17 cells using the flow cytometry analyses, which is a limitation of our study. Actually, the Treg and Th17 cells could be simply verified by analyzing the geometric mean fluorescence intensity (MFI) of FoxP3 and IL-17 or other markers in cells. Second, the CD3^+^CD4^−^ cells (which mainly CD8+ and gamma-delta T (Tgd^+^) cells) can also produce the IL-17 as much as CD3^+^CD4^+^ cells do. However, this study has not provided evidence that CD8+ and Tgd^+^ cells play any roles in the pathogenesis of the ALI by producing the IL-17. In the following study, we would verify the roles of CD8+ and Tgd^+^ cells in ALI pathogenesis. Third, the sample size of ALI model mouse is relatively small. We would involve more large sample size for the animal model in a future study.

## 5. Conclusion

Collectively, our findings showed that BMSCs transplantation could effectively attenuate pathological injury of LPS-induced ALI in mice, which at least partly may be associated with modulation of Treg/Th17 balance and promoted expression of RvE1 and ProD1.
